# ICT Self-efficacy scale: the correlations with the age of first access to the internet, the age at first ownership of a personal computer (PC), and a smartphone

**DOI:** 10.1080/10872981.2022.2151068

**Published:** 2022-11-28

**Authors:** Ziyi Li, Tianming Zuo, Xiaotong Wei, Ning Ding

**Affiliations:** Institute for International Health Professions Education and Research, China Medical University, Shenyang, Liaoning, China

**Keywords:** Information and communication technology (ICT), ICT Self-efficacy, medical students, scale development, psychometric properties, technology experience

## Abstract

**Background:**

Because of the wide use of information and communication technologies (ICT) in healthcare, medical students’ knowledge and skills of modern ICT have been considered essential for their successful learning and future careers. According to Bandura’s self-efficacy, enhancing ICT self-efficacy, which might be affected by technology experience, could be a pathway to improving ICT literacy and competence, which should be one focus of medical educationalists. However, there is a lack of suitable measurements of medical students’ self-efficacy and a clear understanding of its relationship with technology experience.

**Materials and methods:**

We conducted a literature review and direct consultation with an expert panel to identify potential items for the ICT self-efficacy scale. Based on the data collected in a survey of 486 first-year medical students in China, the exploratory factor analysis (EFA) was employed to confirm the structure of the final version. Furthermore, we used linear regressions to quantify the association between ICT self-efficacy and technology experience measured by the age of first access to the Internet, the age at first ownership of a personal computer (PC) or a laptop, and that of a smartphone.

**Results:**

The EFA results derived 15 items of four factors, with 67.02% of the total variance explained: *Privacy and Safety, Differencing, Communication*, and *Learning and Application*. The Cronbach’s alphas for the four subscales and the overall scale ranged from 0.78 to 0.89. Regression results demonstrated a significant association of ICT self-efficacy with age at first ownership of a personal computer (PC) and the mediation role of the general self-efficacy in the ICT self-efficacy’s association with the age at first ownership of a personal smartphone.

**Conclusion:**

The ICT scale developed is a reliable and valid task-specific measure to assess ICT Self-Efficacy for medical students. In addition, enhancing students’ technology experience might improve their ICT self-efficacy.

## Introduction

As information and communications technology (ICT) has grown exponentially, more and more ICT is utilized in various healthcare fields, such as electronic medical records, telemedicine, and decision support tools for healthcare professionals [1,2]. These successful ICT applications improved access to health services, quality and safety of care, continuity of health services, and economic efficiency [3,4]. Therefore, the competence to use ICT efficiently and independently has been considered a critical prerequisite for successfully integrating medical students into the community of health professionals [5–7]. Furthermore, vast amounts of accumulating evidence reveal that trainees and educators utilized ICT to cope with the challenges caused by the pandemic, such as education delivery [8,9], upholding equity, diversity, and inclusion (EDI), medical students on the frontline [10], testing and evaluation [11], confirming the demanding requirement of medical students’ knowledge and skills of modern ICT.

Considering that ICT evolution speeds up so rapidly, rather than specific skills which are outdated very soon, it might be effective and efficient to cultivate medical students’ ICT self-efficacy. According to Bandura’s definition [12], self-efficacy is the belief that a person can execute courses of action required to deal with prospective situations. Furthermore, researchers proved that the self-efficacy of specific domains might significantly predict behavior change and performance in the corresponding domains [13]. Therefore, enhancing medical students’ ICT self-efficacy might improve their ICT competence and academic performance, then competence in healthcare delivery in the digital age, which should be a key focus of contemporary medical schools.

As research suggested, besides formal education in schools, medical students can also acquire and improve their ICT self-efficacy in informal learning at home or in other out-of-school situations [14,15]. Furthermore, as access to ICT technology, including the Internet, personal computers, and smartphones, became quite common in many countries [16], current medical students probably started to use digital technology at very young ages [17]. It is self-evidenced that the new generation of medical students is much better than their elderly peers in ICT self-efficacy on average, providing convenience to the analysis of how the use of ICT at an early age affects the development of ICT self-efficacy in one’s later life. However, research in this field is far from enough, partially because of the lack of applicable measures of ICT-related self-efficacy. Although a small number of scales, for example, Musharraf and her colleagues’ ICT Self-Efficacy scale, had been developed and validated [18], some failed to include typical and fundamental technological applications for students’ online survival in the digital age.

In this study, we would like to develop and validate a task-specific measure of ICT self-efficacy tailored to assess the relationship between medical students’ ICT self-efficacy and when they started using ICT. Items were drafted by considering the implications of Bandura’s self-efficacy theory and ICT skills that current university students apply when achieving their daily tasks. The study’s second objective is to examine the relationship between the first ages medical students’ access to the Internet, possession of their personal computer (PC) and smartphones, and their ICT self-efficacy in their first year of university.

## Materials and methods

### Item pool and content validity

A three-step process was employed to ensure that the specific set of items measuring ICT self-efficacy should be a subset of appropriate items [19]:

- Based on a literature search of existing questionnaires, we identified relevant domains of ICT self-efficacy and created an initial battery of items.
- After consulting with an expert panel of medical education, we established a conceptual framework of ICT self-efficacy with relevant domains. Furthermore, we refined the initial battery of survey items to reflect this underlying construct.
- We reviewed the wording of items to ensure the appropriateness of the content, language level, type and form, and sequence to finalize an item pool for ICT self-efficacy inclusive of 24 items.

Each item was scored from 0 to 4 points on a Likert-type scale, with the highest score indicating greater self-efficacy (1 = Strongly disagree, 2 = Disagree, 3 = Agree, and 4 = Strongly agree) (See Table 1).

**Table 1.** Item pool and structure of ICT Self-efficacy Scale.

Index	Item	Whether in the final version (Y/N)
*Domain: Privacy and Safety*		
1	I can easily hide any activities marked or shared by others on my personal webpage on my most used social networking sites (i.e., Facebook, Weibo, WeChat, QQ, Twitter, Skype, etc.).	Y
2	I can easily block or restrict anyone on my most used social networking sites or apps (i.e., Facebook, Weibo, WeChat, QQ, Twitter, Skype, etc.).	Y
3	I can easily dissolve friendships with anyone on my most used social networking sites or apps (i.e., Facebook, Weibo, WeChat, QQ, Twitter, Skype, etc.).	Y
4	I can easily set a password or PIN code (an identification technology) on my cell phones for security issues.	N
5	I can easily change the password of the email or social networking account that I often use.	Y
6	I can easily report any fake account that claims to be mine.	N
7	I can easily report any ID, post, image, or video as spam or abusive content on my most used social networking sites or apps (i.e., Facebook, Weibo, WeChat, QQ, Twitter, Skype, etc.).	N
8	I can easily change privacy settings on my most used social networking sites or apps (i.e., Facebook, Weibo, WeChat, QQ, Twitter, Skype, etc.).	N
9	I can easily recover my email or social networking account if I forget my password.	N
*Domain: Differencing*		
10	I can easily deal with spam messages in emails or on social networking sites (i.e., Facebook, Weibo, WeChat, QQ, Twitter, Skype, etc.).	Y
11	I can easily judge whether or not the message posted by other people on social networking sites is correct.	Y
12	I can easily identify which information on social networking sites is trustworthy.	Y
13	I am fully aware of the possible consequences of my actions on the Internet.	N
*Domain: Communication*		
14	I can express my point of view in any online discussion forum.	Y
15	I can easily learn its features and functions in a short time when I open any social networking site (i.e., reply, like, etc.).	N
16	I can easily use chat rooms on the Internet.	Y
17	I can easily use the webcam to chat with others.	Y
18	I can easily communicate with friends in a social media group (i.e., WeChat group, QQ group, WhatsApp group, etc.)	Y
*Domain: Learning and Application*		
19	I can easily edit and modify any picture using software on my computer or cell phone (i.e., photoshop, meitu, Snapseed, etc.).	Y
20	I can easily express my point of view using emojis or pictures when chatting with friends online.	N
21	I can easily find digital resources that interest me if I want to.	N
22	If I try my best, I can always find the right software, App, etc., or program it myself to solve the problem.	Y
23	I can easily make and edit short videos using my computer or mobile phone.	Y
24	If I want, I can learn a language and do programming development.	Y

Please note that the English version of the ICTSE scale is not validated.

### Survey and data collection

A cluster random sampling technique was employed. Among the first-year students enrolled in clinical medicine programs of China Medical University in 2021, we randomly selected 26 out of 48 classes. And 650 students from these classes were asked to participate in the survey voluntarily between 1 September 2021, and 30 September 2021. The online survey was constructed and administered using a Wenjuanxing e-questionnaire platform (Wenjuanxing Tech Co. Ltd, Changsha, China) [20], widely used in China. The further exclusion of questionnaires with too many missing values led to an effective response rate of 74.77% (486/650), which is acceptable.

The questionnaire consisted of three parts and included 41 questions. Part I (7 questions) was to collect information on age, gender, the age of first access to the Internet, the age at first ownership of a personal smartphone, whether possessing a smartphone now, the age at first ownership of a personal computer (PC) or a laptop, and whether possessing a computer or laptop now. Part II consisted of the 24 items from the ICT self-efficacy item pool. The final part is the 10-item General Self-Efficacy Scale (GSE) to assess students’ beliefs about coping with difficult life situations.

### Statistical analysis

All statistical analysis was carried out using Stata 14 (Stata- Corp 2015, Stata Statistical Software, Release 14, Stata- Corp, LLP, College Station, Texas, USA). The continuous variables were expressed as Mean ± SD, while categorical data were presented as frequency and percentage. A p-value of <0.05 was considered statistically significant.

## Reliability

We calculated Cronbach’s alpha to test the interitem reliability of the scale. A value of 0.70 or higher was considered acceptable [21].

## Construct validity

Before scale validation, we carried out the Kaiser-Meyer-Olkin (KMO) test to verify the sampling adequacy for the factor analysis and Bartlett’s test of sphericity to indicate sufficient correlations between items.

The construct validity was assessed using principal component analysis (PCA) with an Oblimax rotation technique. We also employed the Promax rotation technique to test whether the possible correlations among factors significantly changed the structure. The number of factors was determined based on the number of eigenvalues above one and the scree plot. Factor loadings equal to or greater than 0.5 were considered appropriate.

## Multiple linear regression

Multiple linear regression analyses were performed to evaluate the possible relationships between ICT self-efficacy and its influencing factors, such as age, gender, and access to the Internet and smartphone. In addition, the potential mediation effect of general self-efficacy in the relationship between ICT self-efficacy and ICT experience was investigated by using the Sobel test [22].

### Ethics approval

Ethics approval was obtained from the Medical Research Ethics Committee of China Medical University before the commencement of the study.

## Results

### Participant characteristics

A total of 486 first-year medical students participated in the study. Of all participants, the mean age was 18.16 years (SD = 0.75);45.47% were male (Panel A, Table 2). Averagely, participants got access to the Internet for the first time at age 9.33 (SD = 2.62) and possessed their first smartphones at age 14.40 (SD = 2.76). Only one student did not have a smartphone. The male students were significantly earlier to access the Internet than females; however, they almost possessed their smartphones at the same age. In terms of general self-efficacy, the average score is 28.08 (SD = 6.52), and female medical students have lower scores than males (26.97 vs. 29.41, P < 0.001).

**Table 2.** Summary statistics of the samples.

Variables	All (N = 486) Mean (SD)	Female (n = 265) Mean (SD)	Male (n = 221) Mean (SD)
Panel A			
Age	18.16(0.75)	18.15(0.77)	18.17(0.74)
Age of first access to the Internet	9.33(2.62)	9.75(2.60)	8.82(2.57)
First-time smartphone ownership	14.40(2.76)	14.27(2.67)	14.55(2.85)
First-time computer ownership	17.15(2.35)	17.35(1.99)	16.92(2.71)
General Self-efficacy	28.08(6.52)	26.97(6.55)	29.41(6.24)
Panel B			
ICT Self-efficacy	42.24(9.27)	41.25(9.21)	43.43(9.23)
Privacy and Safety	12.01(3.47)	11.75(3.39)	12.32(3.55)
Differencing	8.62(2.28)	8.27(2.17)	9.04(2.34)
Communication	11.35(3.16)	11.02(3.22)	11.74(3.04)
Learning and Application	10.27(3.08)	10.21(3.10)	10.34(3.07)

### Scale validation

The value of Kaiser–Meyer–Olkin is 0.88, indicating the sampling adequacy for factor analysis. Bartlett’s Test of Sphericity ($\chi^{2}$=3305.57, p < 0.001) provides additional evidence of the factorability of the data.

The factor loadings by PCA showed that Items 7, 8, 9, 13, 15, and 21 had large positive loadings ≥0.40 on more than two factors; Item 20 seemed irrelevant to any factor; Item 4 and 6 had disproportional low loading compared with the items of the same factor. Additionally, an apparent floor effect for Item 4 was found, with only 1.65% of Chinese medical students feeling it difficult to set the password or PIN on their cellphones.

Dropping all those items above, a rerun of PCA confirmed a structure with four underlying domains (or factors): *Privacy and Safety, Differencing, Communication*, and *Learning and Application*, which is consistent with the suggested structure of the expert panel. The domains, *Privacy and Security*, and *Communication* are about perceived capabilities for personal control and basic behaviors on the Internet. *Differencing* refers to the belief in one’s capacity for information evaluation. The final domain, *Learning and Application*, indicates one’s perceived capabilities of learning and applying what has been learned to solve problems.

The eigenvalues equal 5.89, 1.80, 1.29, and 1.08 for the four factors. The factors explained 39.29%, 11.97%, 8.59%, and 7.17% of items’ variance, respectively, and 67.02% cumulatively. Factor loadings for the 4-Factor model ranged from 0.57 to 0.89 (Table 3). For the final version scale of 15 items, Cronbach’s alpha was 0.89, confirming that a large proportion of the scale’s total variance was attributed to a common source. The Cronbach coefficient alpha for each factor varied from 0.78 to 0.84, indicating the reliability within each domain.

**Table 3.** Factor analysis of ICT Self-Efficacy Scale (N = 486).

Index	Items	Factor Loadings
Cronbach Alpha for the ICT self-efficacy scale (α=0.89)		
*Domain: Privacy and Safety (*α=0.84)		
1	I can easily hide any activities marked or shared by others on my personal webpage on my most used social networking sites (i.e., Facebook, Weibo, WeChat, QQ, Twitter, Skype, etc.).	0.76
2	I can easily block or restrict anyone on my most used social networking sites or apps (i.e., Facebook, Weibo, WeChat, QQ, Twitter, Skype, etc.).	0.88
3	I can easily dissolve friendships with anyone on my most used social networking sites or apps (i.e., Facebook, Weibo, WeChat, QQ, Twitter, Skype, etc.).	0.84
5	I can easily change the password of the email or social networking account that I often use.	0.68
*Domain: Differencing (*α=0.82)		
10	I can easily deal with spam messages in emails or on social networking sites (i.e., Facebook, Weibo, WeChat, QQ, Twitter, Skype, etc.).	0.67
11	I can easily judge whether or not the message posted by other people on social networking sites is correct.	0.87
12	I can easily identify which information on social networking sites is trustworthy.	0.89
*Domain: Communication (*α=0.80)		
14	I can express my point of view in any online discussion forum.	0.63
16	I can easily use chat rooms on the Internet.	0.80
17	I can easily use the webcam to chat with others.	0.72
18	I can easily communicate with friends in a social media group (i.e., WeChat group, QQ group, WhatsApp group, etc.)	0.69
*Domain: Learning and Application (*α=0.78)		
19	I can easily edit and modify any picture using software on my computer or cell phone (i.e., photoshop, meitu, Snapseed, etc.).	0.67
22	If I try my best, I can always find the right software, App, etc., or program it myself to solve the problem.	0.57
23	I can easily make and edit short videos using my computer or mobile phone.	0.77
24	If I want, I can learn a language and do programming development.	0.74

The results confirmed that the ICT Self-Efficacy Scale consisted of four conceptually and statistically validated components. Panel B of Table 2 presents descriptive statistics of ICT self-efficacy, whose average was 42.24 and standard deviation was 9.27. The scores of female students were significantly lower than those of males (41.25 vs. 43.43, P = 0.01). This gender difference mainly stemmed from domain *Differencing* (8.27 vs. 9.04, P < 0.001) and *C*ommunication (11.02 vs. 11.74, P = 0.013).

### The associations of ICT self-efficacy with ICT experience

The ICT self-efficacy of medical students was robustly associated with the age of medical students at first to access the Internet and the first ages of ownership of smartphones and computers. The results of the multiple linear regression adjusted for age and gender show that one year later access to the Internet, ownership of a smartphone, and a PC or laptop were associated with a significant decrease in ICT self-efficacy valued at 0.52 (SE = 0.16), 0.62 (SE = 0.15) and 0.87 (SE = 0.18) respectively (Col.1–3 in Table 4). When all these three variables were introduced into the regression simultaneously, all estimated effects decreased; however, still significant (Col.4 in Table 4).

**Table 4.** Linear regression analysis results to examine the associations between ICT experiences and ICT self-efficacy, and the mediating role of general self-efficacy.

	ICT Self-efficacy				General Self-efficacy	ICT Self-efficacy
Variables	(1)	(2)	(3)	(4)	(5)	(6)
Age of first access to the Internet	−0.52*** (0.16)			−0.30* (0.17)	−0.15 (0.12)	−0.18 (0.13)
First-time smartphone ownership		−0.62*** (0.15)		−0.39** (0.16)	−0.29** (0.11)	−0.15 (0.13)
First-time computer ownership			−0.87*** (0.18)	−0.67*** (0.18)	−0.15 (0.13)	−0.54*** (0.15)
General Self-efficacy						0.83*** (0.05)
Female	−1.70** (0.85)	−2.36*** (0.83)	−1.81** (0.82)	−1.72** (0.83)	−2.31*** (0.59)	0.21 (0.68)
Age	−0.38 (0.56)	−0.35 (0.56)	−0.15 (0.56)	0.19 (0.56)	0.10 (0.40)	0.11 (0.45)
Constant	55.03*** (10.02)	58.84*** (9.94)	60.83*** (9.89)	59.73*** (9.83)	35.71*** (6.99)	29.95*** (8.14)
Observations	486	486	486	486	486	486
*Adj R^2^*	0.04	0.05	0.06	0.09	0.06	0.40

Standard errors in parentheses;*** p < 0.01, ** p < 0.05, * p < 0.1.

**Figure 1.**
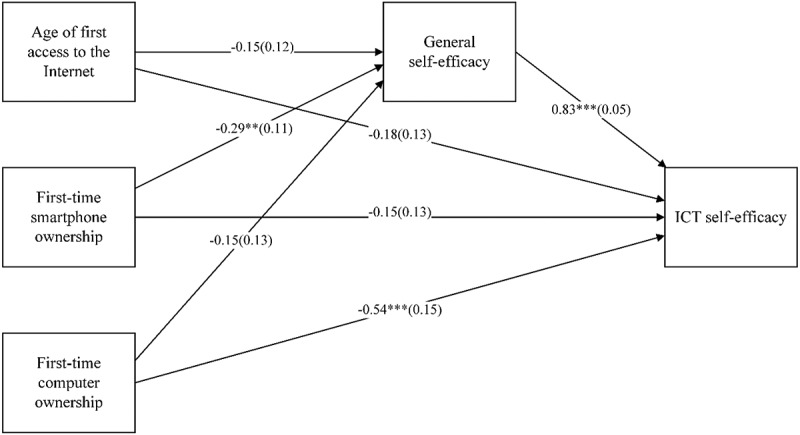
Mediation analysis of General self-efficacy on the association between ICT experience and ICT self-efficacy. (Standard errors in parentheses; *** p < 0.01, ** p < 0.05, * p < 0.1).

Bandura’s self-efficacy theory indicates that general self-efficacy might be correlated with ICT experience and ICT self-efficacy simultaneously, which could lead to a mediation role of general self-efficacy. Col. 5 of Table 4 presented that general self-efficacy was significantly negatively associated with the age at first smartphone ownership among Chinese medical students. However, earlier Internet access or a PC or laptop could not significantly enhance general self-efficacy. After controlling for general self-efficacy, the regression results in Col. 6 demonstrated that general self-efficacy was significantly associated with ICT self-efficacy. Only the age at first ownership of a personal computer (PC) remained to correlate to ICT self-efficacy significantly. Therefore, we might conclude that when the general self-efficacy mediated the association between the age at first ownership of a personal computer or laptop and the ICT self-efficacy, the first computer or notebook ownership might still be directly associated with the ICT self-efficacy. The integration of Col. 5 and 6 of Table 4 is the Sobel test (see Figure 1).

In addition, by introducing the same controls as Col. 6 of Table 4, the possible enhancement effect of possessing a computer or notebook was estimated for each domain of ICT self-efficacy (See Table 5). The results indicate that this estimated enhancement effect was robustly observed for all domains and particularly significant for *Privacy and Safety*, and *Learning and Application*: −0.18 (SE = 0.07) and −0.20 (SE = 0.05), respectively.

**Table 5.** Linear regression analysis results to examine the associations between ICT experiences and each domain of ICT self-efficacy.

	Privacy and Safety	Differencing	Communication	Learning and Application
Variables	(1)	(2)	(3)	(4)
Age of first access to the Internet	0.01 (0.06)	−0.08** (0.04)	−0.05 (0.05)	−0.06 (0.05)
First-time smartphone ownership	−0.07 (0.06)	0.03 (0.03)	−0.01 (0.05)	−0.10** (0.04)
First-time computer ownership	−0.18*** (0.07)	−0.07* (0.04)	−0.10* (0.06)	−0.20*** (0.05)
General Self-efficacy	0.15*** (0.02)	0.18*** (0.01)	0.24*** (0.02)	0.26*** (0.02)
Female	−0.16 (0.31)	−0.21 (0.18)	−0.05 (0.26)	0.63*** (0.23)
Age	−0.42** (0.20)	0.08 (0.12)	0.02 (0.17)	0.42*** (0.15)
Constant	19.41*** (3.68)	3.54 (2.15)	6.59** (3.08)	0.41 (2.78)
Observations	486	486	486	486
R-squared	0.14	0.31	0.27	0.38

Standard errors in parentheses; *** p < 0.01, ** p < 0.05, * p < 0.1.

## Discussion

The key findings of the present study are as follows: 1) The ICT self-efficacy scale contained four domains: *Privacy and Safety, Differencing, Communication*, and *Learning and Application*, which was a valid tool for Chinese medical students. 2) the ICT experience measured by the first age of accessing the Internet, the age at first ownership of a personal computer or a laptop, and the age at first ownership of a smartphone was significantly associated with ICT self-efficacy in Chinese medical students. 3) The general self-efficacy significantly mediated the estimated effect of age at first ownership of a personal smartphone on ICT self-efficacy. 4) The age at first ownership of a PC or laptop was associated with each domain of ICT self-efficacy, particularly significantly with *Privacy and Safety*, and *Learning and Application*.

Bandura defined *self-efficacy* as people’s judgments of their capabilities [23] to organize and execute courses of action required to attain designated types of performances, which guided the scale development in this study. Since individuals’ efficacy judgments vary with specific goals, self-efficacy should be domain- and task-specific. As a result, to assess self-efficacy, educational research often asks participants to rate the strength of their belief in their ability to execute the requisite activities [24]. However, if researchers fail to measure self-efficacy correctly due to their misunderstanding of the construct, the assessments usually have poor predictive power [25]. Thus, in this study, we firstly invited a panel of experts in medical education to clarify the construct of ICT self-efficacy, resulting in a 4-domain structure of ICT self-efficacy. Then, by considering the current ICT development situation in ICT-developed countries like China [26,27], we specified the tasks for each domain and finalized the items. Therefore, the construct of the ICT self-efficacy scale could be practicable in other countries or regions; however, the items may need to be revised according to regional economic and social situations, such as the prevalence of PCs and smartphones and the construction of telecommunications networks.

The regression analysis in this study focused on the relationship between the age when medical students access the Internet, possessing a smartphone or a PC or a laptop, and their ICT self-efficacy. Following the previous findings in the literature, these relationships are expected to be negative, i.e., the earlier one accesses the Internet, ownership of a smartphone or a computer, the higher level of ICT self-efficacy [28,29]. These hypotheses are confirmed in almost all the regressions, even when controlling for student gender, age, and general self-efficacy. Compared with the work of Hatlevik and his colleagues, which achieved a similar result, this study used a nuanced marker for experience with technology (i.e., three variables of first ages) [29]. When introducing all these three variables simultaneously into the regression, the estimated effect of the age at first ownership of a personal computer (PC) or a laptop was the most significant; however, those of Internet and smartphone were only marginally significant.

So, when considering the possible correlations among the different technology experiences, computer usage might be the most effective way to enhance one’s ICT self-efficacy. And in addition, considering the well-observed correlation between ICT self-efficacy and skills, it could be also effective in cultivating one’s ICT skills [15,30].

As Sherer et al. stated, one’s past experiences with success and failure in various situations could result in expectations that one carries into new situations [31]. In this way, the experience of specific tasks could enhance general self-efficacy; meanwhile, general self-efficacy might also improve the efficacies of certain domains or tasks reversely. Therefore, general self-efficacy could work as a mediator of technology experience. The mediation effect analysis in this study verifies this hypothesis. The results demonstrate that among Chinese medical students, general self-efficacy behaved quite differently in its mediation effect on the relationships between the three types of technology experience and ICT self-efficacy:

- When taking general self-efficacy as the mediator, the age of first access to the Internet had no direct or indirect effect on ICT self-efficacy, consistent with the marginal significance of the estimated overall effect.
- Although the smartphone experience seemed to have no direct effect on ICT self-efficacy, general self-efficacy worked as an essential mediator in this relation resulting in a significant indirect effect.
- The estimated direct effect of the computer possesses on ICT self-efficacy was very significant, and there was no mediation effect of general self-efficacy.

Further analysis of the association between technology experiences and the domains of ICT self-efficacy might explain the findings above. The estimated direct effect of the age of first access to the Internet is marginally significant for *Differencing* only. In fact, for current medical students in China who were born in a digital world, the experience of surfing the Internet is pervasive because of the prevalence of network devices and PCs. However, when facing massive information from the Internet, they always need to distinguish valuable information from junk information, which develops their capacity for information differencing and improves their belief on such capacity constantly. Furthermore, for current medical students, PCs and smartphones are not only tools for communication and entertainment but also learning; thus, both PC and smartphone experiences directly affect students’ beliefs on *Learning and Application*. Considering the broader use of PCs in problem-solving, it is not surprising that the estimated effect of PC experience was more significant than those of Internet surfing or smartphone usage.

A limitation of this study is that a cross-sectional observational cohort from one medical university was utilized for initial validation and subsequent regression analysis. Since the validation is in a single institute setting, bias may increase along with limitations of generalizability. The future study might focus on the generalization pattern of this scale by implementing multi-institute surveys. Furthermore, although we controlled for age and gender, the information on students’ socioeconomic backgrounds was not collected and so was not introduced into regressions. However, such background information is vital for understanding variations in students’ ICT self-efficacy because it may explain digital inequity and the digital divide. Thus, we suggest future studies on ICT self-efficacy or technology experience collect such information. In addition, this study will hopefully motivate further research on the development of the ICT self-efficacy scale regarding the relationship between personal characteristics, background contextual variables, and ICT self-efficacy.

In conclusion, we have developed and validated a novel tool to assess ICT self-efficacy. Furthermore, we propose that individuals’ technology experiences, including internet access, and the utilization of PCs and smartphones, are significant predictors of their ICT self-efficacy. Therefore, to help medical students follow the trends of educational reforms driven by ICT development trends, schools and families should provide opportunities to enhance students’ technology experience and ICT self-efficacy.

## Data Availability

The data supporting this study’s findings are available from the corresponding author, ND, upon reasonable request.
